# A comprehensive database of Nature-Inspired Algorithms

**DOI:** 10.1016/j.dib.2020.105792

**Published:** 2020-06-02

**Authors:** Alexandros Tzanetos, Iztok Fister, Georgios Dounias

**Affiliations:** a Management and Decision Engineering Laboratory, Department of Financial and Management Engineering, School of Engineering, University of the Aegean, Greece; b University of Maribor, Faculty of Electrical Engineering and Computer Science, Institute of Informatics, Maribor, Slovenia

**Keywords:** Nature-inspired algorithms, Swarm intelligence, Nature-inspired computing, Bio-inspired algorithms, Evolutionary algorithms, Evolutionary computing

## Abstract

These data contain a comprehensive collection of all Nature-Inspired Algorithms. This collection is a result of two corresponding surveys, where all Nature-Inspired Algorithms that have been published to-date were gathered and preliminary data acquired. The rapidly increasing number of nature-inspired approaches makes it hard for interested researchers to keep up. Moreover, a proper taxonomy is necessary, based on specific features of the algorithms. Different taxonomies and useful insight into the application areas that the algorithms have coped with is given through these data. This article provides a detailed description of the above mentioned collection.

**Specifications Table**

**Table:** 

**Subject**	Artificial Intelligence
**Specific subject area**	Nature-Inspired Algorithms
**Type of data**	csv file
**How data were acquired**	Data were acquired through research in documents and records from International Journals and Conferences.
**Data format**	**Raw:** csv file
**Parameters for data collection**	Only Nature-Inspired Algorithms are included in this dataset, based on the definition given by [1]: “The term nature refers to any part of the physical universe which is not a product of intentional human design”. To select the algorithms meeting the above definition properly, the authors read the initial study proposing the algorithm and excluded methods inspired by social theory (Political Optimiser, etc.), sports (i.e. the League Championship Algorithm) or the result of intentional human design (such as the Fireworks Algorithm).
**Description of data collection**	These data were collected through web research.
**Data source location**	Worldwide
**Data accessibility**	Repository name: Mendeley Data identification number: 10.17632/xfnzd2c8v7.1 Direct URL to data: http://dx.doi.org/10.17632/xfnzd2c8v7.1

**Value of the Data**

• These data consist of the first comprehensive list of Nature-Inspired Algorithms, where the main information for each algorithm can be found (year, authors, Journal or Conference where it was initially presented, applications that were tackled in the initial work, etc.). Moreover, information is included on the application areas that each algorithm has been applied to.
• Interested audiences can benefit from this data set, while also, researchers who are interested in narrowing down their choices when trying to find a proper algorithm for their application. Furthermore, the algorithms included in this database will benefit and be introduced to a greater number of readers.
• Useful insights can be extracted from these data. Based on this data set, more secondary data could be carried out that will lead to adequate survey studies.
• Furthermore, the field of Nature-Inspired Intelligence would benefit from this data set. New hybrid schemes could be developed based on the provided information of the data, while also further research can be done on the features that an algorithm should have to cope with a specific problem or problem area.
• Finally, the provided data set could even be used as a benchmark for future surveys that focus on a specific application area. Additionally, these data also allow the citation and bibliometric analysis of papers in the area of Nature-Inspired Computing.

## 1 Data description

The data described in this article consist of all Nature-Inspired Algorithms that have been published to-date. To define which meta-heuristics can be considered Nature-Inspired, the definition given by [1] is used, stating that “the term nature refers to any part of the physical universe which is not a product of intentional human design”.

The database consists of 43 variables, as follows:

The data include the algorithm's name (variable 1), the abbreviation (variable 2), the year presented (variable 3), the authors (variables 6–14, where applicable), the Journal or Conference where the algorithm was published initially (variables 15–16), and the applications tackled in this initial work (variables 19–22) (Table 1).

**Table 1:** Description of data set attributes.

**N.**	**Attribute**	**Format**	**Description**	**Values**
1	Algorithm_name	Text	The algorithm's name	
2	Abbr.	Text	Abbreviation of the algorithm	
3	Year	Date (yyyy)	Year presented	
4	Category	Categorical	The Nature-Inspired intelligent category to which the algorithm belongs	1–3
5	Subcategory	Categorical	The sub-category of the above main category	11–33
6	Author1	Text	First author of initial work presenting the algorithm	
7	Author2	Text	Second author of initial work presenting the algorithm	
8	Author3	Text	Third author of initial work presenting the algorithm	
9	Author4	Text	Next author of initial work presenting the algorithm	
10	Author5	Text	Next author of initial work presenting the algorithm	
11	Author6	Text	Next author of initial work presenting the algorithm	
12	Author7	Text	Next author of initial work presenting the algorithm	
13	Author8	Text	Next author of initial work presenting the algorithm	
14	Author9	Text	Next author of initial work presenting the algorithm	
15	Publication	Categorical	Where was the algorithm presented initially (Journal or Conference)?	1–2
16	Jrnl_or_Conf_name	Text	Name of the Journal or the Conference	
17	Publisher	Categorical	Under which publication house was the algorithm published initially?	1–50
18	Application	Categorical	Has the algorithm been applied in real problems in the initial work?	0–2
19	App1	Categorical	First application of the algorithm in initial work	1–54
20	App2	Categorical	Second application of the algorithm in initial work	1–54
21	App3	Categorical	Third application of the algorithm in initial work	1–54
22	App4	Categorical	Fourth application of the algorithm in initial work	1–54
23	Notes	Categorical	Notes on the paper	1
24	EngTotal	Numeric	# of published works in Engineering Applications	
25	EngJournals	Numeric	# of Journal publications in Engineering Applications	
26	FinTotal	Numeric	# of published works in Finance Applications	
27	FinJournals	Numeric	# of Journal publications in Finance Applications	
28	OR_Total	Numeric	# of published works in Operational Research Applications	
29	OR_Journals	Numeric	# of Journal publications in Operational Research Applications	
30	EnerTotal	Numeric	# of published works in Energy Applications	
31	EnerJournals	Numeric	# of Journal publications in Energy Applications	
32	OtherTotal	Numeric	# of published works in Other Optimisation Applications	
33	OtherJournals	Numeric	# of Journal publications in Other Optimisation Applications	
34	OptTotal	Numeric	# of published works in Optimisation Applications (sum of the previous categories)	
35	OptJournals	Numeric	# of Journal publications in Optimisation Applications (sum of Journal publications of previous categories)	
36	ClustClassTot	Numeric	# of published works in Clustering/Classification Applications	
37	ClustClassJour	Numeric	# of Journal publications in Clustering/Classification Applications	
38	ForecTotal	Numeric	# of published works in Forecasting and other Applications	
39	ForecJour	Numeric	# of Journal publications in Forecasting and other Applications	
40	NoApplicAlg	Categorical	Nominal variable denoting algorithms without any application	0–1
41	Established	Categorical	Established algorithms (with over 200 application publications)	0–1
42	Total_Works	Numeric	Total published implementations	
43	TotalWorksBinned	Categorical	Total published implementations (Binned)	0–5

In variables 4 and 5, the algorithms are categorised based on their main inspiration category and the sub-category, as in [2,3]. The categories and the subcategories are presented in Table 2:

**Table 2:** Categories and subcategories of Nature-Inspired Algorithms.

**Value**	**Category/subcategory**	**Variable**
1	Swarm Intelligence	4
11	Foraging	5
12	Social Behaviour	5
13	Other Swarm Behaviours	5
2	Organisms-based	4
21	Fauna	5
22	Flora	5
23	Other	5
3	Physical Phenomena & Laws of Science	4
31	Universe	5
32	Nature Phenomena	5
33	Laws of Science	5

Variable 15 categorises algorithms based on where they were presented as:

1 Journal
2 Conference/Congress

While, in variable 16, the corresponding Journal or Conference name is given.

The publisher of the Journal or Conference Proceedings is included in variable 17, as is shown in Table 3.

**Table 3:** Value description for variable 17 regarding publication houses.

**Value**	**Description**
1	Springer
2	Elsevier
3	IEEE
4	ACM
5	InderScience
6	Hindawi
7	Wiley
8	World Scientific
9	Taylor & Francis
10	AIP Publishing
11	arXiv.org
12	IOS Press
13	EMW Publishing
14	Publications International
15	Kaunas University of Technology
16	American Physical Society
17	AAAS
18	Islamic Azad University, Rasht Branch
19	Hikari
20	MDPI AG
21	Academic Journals
22	IRAQI Academic Scientific Journals
23	Iran University of Science & Technology
24	Morgan Kaufmann Publishers
25	Foundation of Computer Science
26	Zhejiang University
27	Tsang Hai Book Publishing Co.
28	Emerald Publishing Limited
29	IGI Global
30	Canadian centre of Science and Education
31	bepress
32	University of Essex
33	OMICS International
34	Sharif University of Technology
35	i6doc
36	World Academic Press
37	Medwell Publishing
38	NADIA
39	Institute of Advanced Engineering and Science
40	Science Publishing Corporation
41	Global Trends Academy
42	The Institute of Research & Community Outreach - Petra Christian University
43	International University of Sarajevo
44	Atlantis Press
45	EBSCO Industries
46	Building & Housing Research centre
47	Sage Journals
48	COPPE Publication
49	Scientific Research
50	Linköping University Electronic Press

All applications areas, where at least one algorithm has been applied, are given in Table 4.

**Table 4:** Value description for variables 19–22.

**Value**	**Description**
0	Benchmark functions
1	Design Engineering Optimisation
2	TSP/VRP
3	Knapsack
4	Scheduling Problems
5	Assignment Problems
6	Character/Pattern Recognition
7	Principal Components Analysis
8	Clustering
9	SAT
10	Classification
11	Mobile Network Deployment Problem
12	Design of Off-Shore Wind Farms
13	Placement-Wiring
14	Potential Problems
15	Stable Linear System
16	Artificial Neural Networks` training
17	Graph Colouring
18	Image Tracking
19	TSC problem
20	Cell Formation Problems
21	Hydrogeologic Parameter Estimation Problem
22	Economic Load dispatch
23	Hull-form SBD
24	Graph Partitioning
25	Reliability
26	Quanser Heat Flow Experiment
27	The Steiner problem
28	Motion Estimation
29	Image Thresholding
30	Groundwater Model Calibration
31	Airfoil design
32	Finite Element Inverse Analysis
33	Load Frequency Control
34	Chlorobenzene Purification Process Design
35	Optimal Power Flow
36	Heat Flow Experiment
37	Minimal exposure problem of wireless sensor networks
38	Steiner tree problem
39	Air Robot Path Planning Problem
40	Solar PV Array
41	Optimal Crop Rotation Problem
42	Robust Control Theory
43	Speed reducer problem
44	p-Median Problem
45	Customer Segmentation Problem
46	EMC filter
47	Suspicious Person Detection
48	Robot navigation
49	N-Queens Problem
50	Pattern Recognition
51	Optimisation of Rainbow Boxes
52	Software Development Effort Estimation
53	Fake Review Detection
54	Identification of hydrogeological parameters

Variable 18 provides an algorithm taxonomy based on the application tackled in the initial work, as:

1 No
2 Yes
3 Only Benchmark functions

Notes about the algorithm are included in variable 23. In the initial version of the dataset only one algorithm has a note, which initial work has been retracted. This note has taken the value of 1, and in future versions of the data set, more values would be added if applicable.

Furthermore, the data include the number of published papers in five optimisation problem areas, i.e. engineering problems (variables 24–25), financial problems (variables 26–27), operations research (variables 28–29), energy problems (variables 30–31) and other optimisation problems (variables 32–33). The total number of all these areas can be seen in the corresponding feature (variables 34–35), As well asapplications of each algorithm in clustering and/or classification problems (variables 36–37), and also forecasting ones (variables 38–39). In all cases, two variables are used, where the first variable of each pair denotes the total number of works, while the second one denotes only the number of works published in Journals.

From the above information, two variables have been added, where total works are calculated (variable 42) and the existence of application is denoted as (variable 40):

1 Algorithms without application
2 Algorithms applied in at least one problem area (without taking into consideration the work where they have been published)

Moreover, another categorical variable (variable 43) denotes the total published works as:

1 No applications
2 <= 50 applications
3 51 - 100 applications
4 101 - 150 applications
5 151 - 200 applications
6 201+ applications

Based on the above classification, a dummy variable (variable 41) has been generated, in which algorithms are classified in:

1 Non-established and
2 Established algorithms, where methods with over 200 applications are included

## 2 Experimental design, materials, and methods

The data described in this article have been acquired from 2017 todate. They are divided into preliminary data acquired through documentation (variables 1–23) and secondary data (variables 24–44), which have been calculated using several scientific repositories.

Initially, based on the work of [4], the authors collected some Nature-Inspired algorithms, where the algorithm's name, the abbreviation, the year presented, the authors, the Journal or Conference where the algorithm was initially published and the applications tackled in this initial work have been noted. IBM's SPSS package was used to organise all these features. Useful information has also been found in [5,6]. This database has been updated on a monthly basis.

Furthermore, the number of papers where each algorithm is applied in various problem areas has been calculated through web research. Using Google Scholar, Mendeley and other scientific repositories, [2,3], we collected the number of published papers in five optimisation problem areas, i.e. engineering problems, financial problems, operations research, energy problems and other optimisation problems. The total number of applications of each algorithm has been calculated in optimisation, clustering and/or classification problems, while also forecasting problems.

The total works have been calculated from the above information, and a binary variable denotes if the algorithms have been applied in at least one problem area, without taking into consideration the work where they were published. Another categorical variable has been added, which performs taxonomy of the algorithms based on the total published works. Based on the number of applications, a dummy variable has been generated, in which algorithms are classified as non-established and established algorithms.

**Fig. 1:**
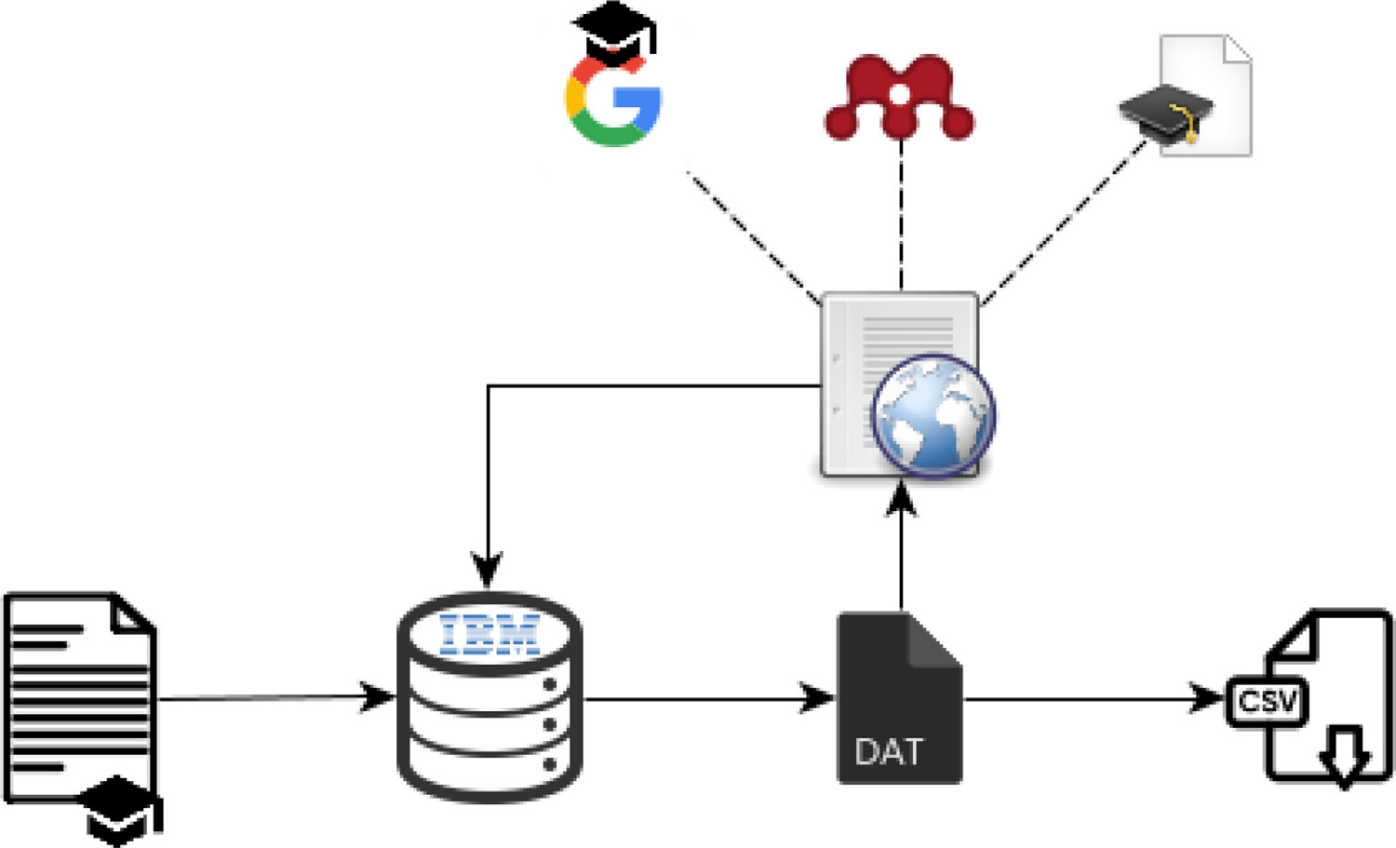
Data acquisition.

The monthly update is performed via web research in scientific repositories, as is shown in Fig. 1. The final database is exported in csv format.

Interested readers can find source codes of some of the corresponding algorithms in libraries such as DEAP [7], NiaPy [8], jMetalPy [9], PySwarms [10], etc.

## Declaration of Competing Interest

The authors declare that they have no known competing financial interests or personal relationships which have, or could be perceived to have, influenced the work reported in this article.

## Appendix Supplementary materials

Image, application 1
